# Role of Ubiquitylation in Controlling Suppressor of Cytokine Signalling 3 (SOCS3) Function and Expression

**DOI:** 10.3390/cells3020546

**Published:** 2014-05-30

**Authors:** Jamie J. L. Williams, Kirsten M. A. Munro, Timothy M. Palmer

**Affiliations:** Institute of Cardiovascular and Medical Sciences, University of Glasgow, Glasgow G12 8QQ, UK; E-Mails: Jamie.Williams@glasgow.ac.uk (J.J.L.W.); k.munro.1@research.gla.ac.uk (K.M.A.M.)

**Keywords:** SOCS3, JAK, STAT, inflammation, ubiquitin, proteasomal degradation

## Abstract

The realisation that unregulated activation of the Janus kinase–signal transducer and activator of transcription (JAK–STAT) pathway is a key driver of a wide range of diseases has identified its components as targets for therapeutic intervention by small molecule inhibitors and biologicals. In this review, we discuss JAK-STAT signalling pathway inhibition by the inducible inhibitor “suppressor of cytokine signaling 3 (SOCS3), its role in diseases such as myeloproliferative disorders, and its function as part of a multi-subunit E3 ubiquitin ligase complex. In addition, we highlight potential applications of these insights into SOCS3-based therapeutic strategies for management of conditions such as vascular re-stenosis associated with acute vascular injury, where there is strong evidence that multiple processes involved in disease progression could be attenuated by localized potentiation of SOCS3 expression levels.

## 1. Introduction

Cytokines control many important biological responses: these include, but are not limited to, haematopoiesis, T cell differentiation and expansion, and inflammatory status. Several cytokine receptors, including gp130 (the signal transducing component of the interleukin (IL)-6 signalling complex), ObRb (leptin receptor) and IFNGR (interferon γ receptor), activate receptor-associated Janus kinases (JAKs) which then trigger the tyrosine phosphorylation and activation of signal transducer and activators of transcription (STATs) and other receptor-interacting proteins, such as SH2 domain-containing protein tyrosine phosphatase (SHP2), to initiate their intracellular effects. Tyrosine-phosphorylated STATs can then dimerise and translocate to the nucleus, where they function as transcription factors by binding to specific promoter elements and recruiting transcriptional co-activators [1,2]. In contrast, SHP2 is primarily responsible for activating the Ras-mitogen-activated/extracellular signal-regulated kinase (MEK)-extracellular signal-regulated kinase (ERK) 1/2 pathway via any one of several proposed mechanisms. Furthermore, SHP2-dependent activation of ERK1/2 also drives Gab1-dependent phosphatidylinositol-3-kinase (PI3K)-Akt signalling, as ERK1/2-dependent phosphorylation of Gab1 on Ser552 initiates its sequestration to the plasma membrane through interaction with phosphatidylinositol-3,4,5-trisphosphate (PIP_3_) via its pleckstrin-homology domain (PH). Gab1 then acts as a scaffold for recruitment of PI3K as well as other signalling components, such as Grb2 and phospholipase C (PLC) γ as well as SHP2 [3].

Multiple temporally distinct inhibitory mechanisms operate at several levels to ensure that signalling responses downstream of activated cytokine receptors are transient in nature. The significance of this has been demonstrated by observations showing that chronic activation of such pathways initiates and perpetuates several chronic inflammatory diseases, such as rheumatoid arthritis and atherosclerosis, as well as haematological malignancies (e.g., polycythemia vera) and solid tumour development (e.g., cholangiocarcinoma) [4,5,6,7]. Negative regulation can occur through both extracellular and intracellular mechanisms. For example, extracellular soluble gp130 (sgp130) can trap circulating soluble IL-6Rα/IL-6 complexes and thus suppress inappropriate *trans*-signalling associated with disease [8]. A particularly important intracellular mechanism is the functional inhibition of signalling complexes following the induction of suppressors of cytokine signalling (SOCS) proteins [9]. In this review, we will focus on the importance of one of the SOCS family members, SOCS3, and the significance of a key aspect of its function as an inducible substrate-binding component within a multi-subunit E3 ubiquitin ligase complex.

## 2. Suppressor of Cytokine Signalling 3 (SOCS3)

SOCS proteins constitute a family of eight related proteins (CIS, SOCS1-7), of which SOCS1 and SOCS3 have been most intensively characterised. These proteins were identified initially by their now well established role as inhibitors of signal propagation from specific cytokine receptors [reviewed in 1,9].

**Figure 1:**
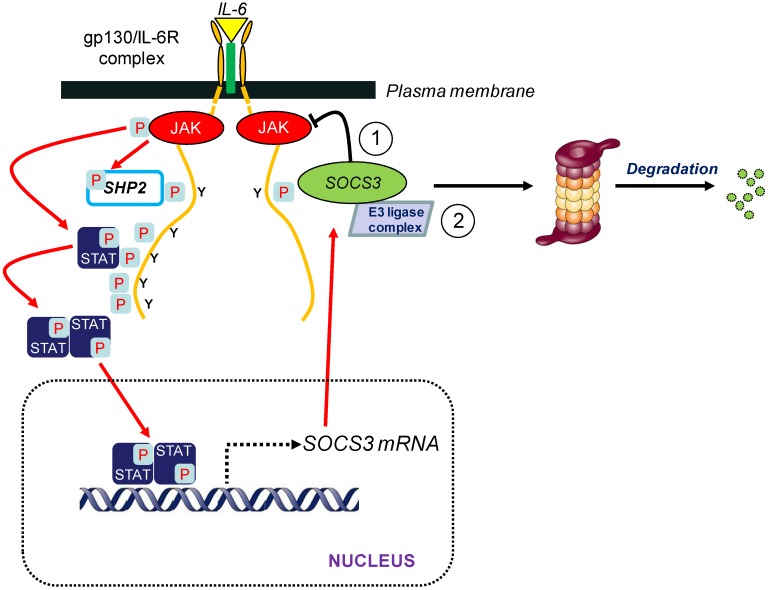
SOCS3-mediated inhibition of IL-6 signalling interaction of IL-6 with a membrane-bound IL-6 receptor (IL-6Rα) and gp130 dimers triggers activation of gp130-bound JAKs, which then phosphorylate gp130 on key cytoplasmic Tyr residues that act as docking sites for SH2 domain-mediated interaction with target proteins. These include four pYXXQ motifs that recruit STAT proteins (predominantly STAT3) and a pY^759^STV (human sequence) motif responsible for binding protein Tyr phosphatase SHP2. Recruited SHP2 and STATs are then phosphorylated by activated JAKs (e.g., STAT3 is phosphorylated on Tyr705). JAK-phosphorylated STATs then dimerise and translocate to the nucleus to initiate transcription of target genes. One of the induced genes encodes SOCS3, which can then interact with Tyr759-phosphorylated gp130 to terminate IL-6 signalling predominantly via two mechanisms; KIR (kinase inhibitory region)-mediated inhibition of receptor-bound JAKs (1), and formation of an E3 ubiquitin ligase complex that ubiquitylates target proteins for subsequent degradation by the proteasome (2).

SOCS proteins function as classical negative feedback inhibitors of cytokine signalling, since most SOCS proteins are themselves cytokine-inducible (Figure 1). Cytokines shown to induce SOCS3 include the gp130 signalling cytokines (e.g., IL-6, oncostatin M), IL-2, IL-3, IL-4, IL-10, type I and type II interferons (IFNs) and leptin as well as Toll-like receptor (TLR) agonists (e.g., lipopolysaccharide (LPS), CpG-DNA), growth hormone (GH), prolactin and cyclic AMP-mobilising hormones [10,11,12,13]. Upon induction, SOCS3 regulates the magnitude, kinetics, and quality of JAK/STAT signalling initiated from multiple receptors. This is mediated by SOCS3 binding to specific PTyr residues on downstream targets via its central SH2 domain (Figure 1). Although SOCS3 binds to the SHP2 binding site PTyr759 (PTyr757 in mouse) on gp130 [14] and has a similar affinity to SHP2 for the target phosphopeptide *in vitro* [15], it is considered unlikely that SOCS3 inhibits ERK1/2 and PI3K activation via direct competition for the SHP2 binding site, as SHP2 and SOCS3 have been shown to act independently to inhibit IL-6 signalling [16].

**Figure 2:**
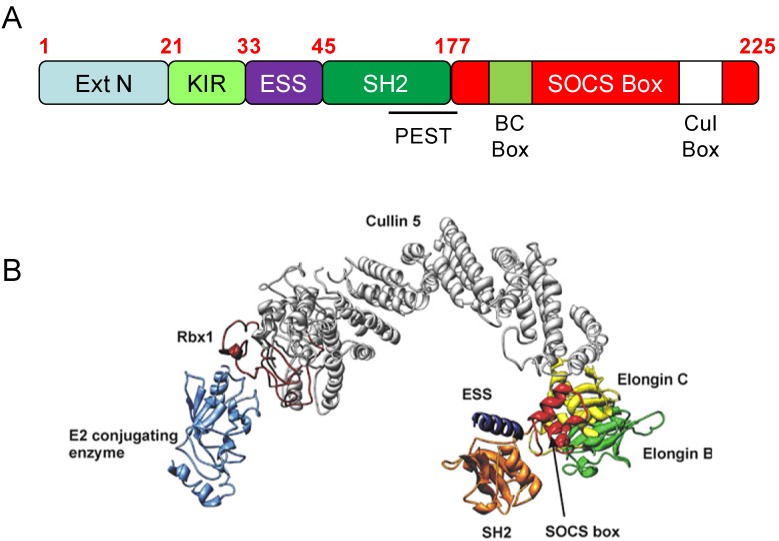
Structural organisation and homology modelling of an ECS^SOCS3^ complex A. Organisation of domains within SOCS3: the numbering is for human SOCS3. Domains include an extended N-terminal region, the kinase inhibitory region (KIR), an extended SH2 sub-domain (ESS) which precedes the central SH2 domain, and the SOCS box, which includes BC box and Cul box sub-domains important for binding the elongins and cullin proteins and forming the E3 ubiquitin ligase complex. Also labelled is a PEST sequence within the C-terminal region of the SH2 domain. B. Structural homology model of the ECS^SOCS3^ E3 ubiquitin ligase complex. The central cullin 5 scaffold protein positions the E2 conjugating enzyme in close proximity to SOCS3, which binds a target substrate (not shown) via its SH2 domain. SOCS3 is attached to cullin 5 both directly (via the Cul box) and indirectly through the elongin B/elongin C heterodimer. The triple α-helical structure of the C-terminal domain SOCS box forms a four-helix bundle with α-helix 4 of elongin C. Reproduced from [28]. You jumped the numbers in between.with permission.

SOCS1/3 contain a unique 12-residue N-terminal kinase inhibitory region (KIR) [17], a pseudosubstrate domain that is capable of interacting with the substrate binding site of the JH1 catalytic domain of receptor-associated JAKs to inhibit substrate phosphorylation (Figure 2). In fact, SOCS1 and SOCS3 are the only SOCS family members able to directly bind JAKs, although they may ultimately regulate Tyr kinase activity via distinct mechanisms [18]. A mechanism of action for SOCS3 has been proposed by Babon and colleagues, who have demonstrated that SOCS3 exerts an inhibitory function on JAK1, JAK2 and Tyk2 but not JAK3 due to the absence of the hydrophobic amino acid sequence (GlyGlnMet or GQM) in JAK3 [19,20]. The GQM sequence, located at positions 1071–1073 on JAK2, is located near the JAK insertion loop, an α-helical region of the JH1 kinase domain that is unique to the JAK family [21], and facilitates the binding of SOCS3 via the extended SH2 subdomain (ESS), SH2 and KIR domain collectively. Interestingly, minimal structural changes were observed in JAK2 following SOCS3 docking at the hydrophobic GQM motif [20]. However, mutation of a key residue within the KIR of SOCS3 (Phe25Ala) results in the loss of inhibitory function by SOCS3, thereby confirming the importance of this domain. In addition, the authors proposed that SOCS3 may act as a pseudosubstrate of JAK2, inhibiting its function by blocking cognate substrates binding [19]. Further supporting the role of the KIR in this process, deletion of the first 3 residues in the KIR results a ten-fold increase in the IC_50_ value for SOCS3 inhibition of JAK2 activity. Moreover, the crystal structure of the SOCS3-JAK2-gp130 complex has revealed that Arg21 within SOCS3, which flanks the KIR, can interact with the JAK2 substrate binding domain and function as part of a pseudosubstrate sequence. This hypothesis was confirmed when mutation of the first 3 residues in the KIR to tyrosine residues led to the phosphorylation of SOCS3 at these sites [20].

## 3. SOCS3 and E3 Ubiquitin Ligase Activity

### 3.1. Introduction

In common with all SOCS family members, SOCS3 shares a SOCS box motif that enables formation of an elongin-cullin-SOCS (ECS) E3 ubiquitin ligase complex that can target bound substrates for Lys48-linked polyubiquitylation and proteasomal degradation. SOCS3 can bind the cullin 5 scaffold protein directly via a Leu^210^ProGlyPro motif within the SOCS box [22] and also viainteraction with an elongin B/C dimer that binds the N-terminal region of cullin 5. Cullin 5 also binds the Really Interesting New Gene (RING) domain-containing protein Rbx2 via its C-terminus, which enables interaction with the E2 conjugation protein [23,24] (Figure 2). However, SOCS3 has an approximately ten-fold lower affinity (K_D_ = 10^−7^ M) for the E3 scaffold components compared to other SOCS family members due to a slower on-rate, and also has a shorter half-life. This reduction in affinity is due to sequence variations within the cullin5 binding site (LeuProGlyPro for SOCS3 *versus* LeuPro*Leu*Pro for all other SOCS proteins apart from SOCS1) [25]. As such, two subclasses of SOCS proteins can be defined based on their differing affinity for cullin5 [25]. Thus, SOCS1 and SOCS3 appear to have dual roles while other SOCS members might regulate signalling exclusively via ubiquitin-dependent pathways. However, while E3 ligase functionality has been demonstrated for SOCS1/3, it has yet to be confirmed for other SOCS family members [25]. The full spectrum of ubiquitin-regulated SOCS1/3 substrates is unknown, but some of those identified thus far are shown in Table 1. Moreover, studies on mice that have been genetically manipulated to remove either the SOCS1 or SOCS3 SOCS box domains have revealed several immunological defects, suggesting proteasome-dependent regulatory roles of both SOCS1 and SOCS3 in controlling such processes [26,27].

**Table 1:** Known SOCS3-dependently ubiquitylated and degraded proteins.

Substrate		Ref.
1	Janus kinase 1 (JAK1)	[29]
2	Focal adhesion kinase 1 (FAK1)	[30]
3	Insulin receptor substrate 1/2 (IRS1/2)	[31,32]
4	Sialic acid-binding Ig-like lectin (SIGLEC) 3/CD33	[33]
5	Sialic acid-binding Ig-like lectin (SIGLEC) 7	[34]
6	Indoleamine 2,3-dioxygenase (IDO)	[35]
7	Granulocyte colony-stimulating factor receptor (G-CSFR)	[36]

Integral to both inhibitory and E3 ligase functionality is the central SH2 domain which allows SOCS proteins to associate with tyrosine-phosphorylated targets (Figure 2). As such, upon formation of the larger E3 ligase complex, it might turn out that steric hindrance prevents SOCS3 binding to specific substrates. It is possible therefore that SOCS3 may exist as two different functional entities *i.e.* an inhibitory protein or as an E3 ligase complex, the balance between which is dictated by the abundance or availability of E3 components. However, it is also possible that SOCS3 can sequentially inhibit and ubiquitylate its substrates meaning that, in certain circumstances, assigning independent roles for each domain might not be possible. While the full impact on individual signalling pathways is not as yet fully appreciated, the following section describes those substrates known to be targeted for ubiquitylation in a SOCS3-dependent manner.

### 3.2. Putative Substrates for SOCS3-Mediated Ubiquitylation

To date, only a handful of SOCS3 substrates have been verified as being ubiquitylated and proteasomally-degraded in a SOCS3 SOCS box-dependent manner (Table 1). However, it should be noted that most of these studies have been performed in cellular overexpression systems, and will ultimately require validation from cell-free ubiquitylation assays using purified components. The substrates identified so far regulate unique cellular functions, but common to each is their Tyr phosphorylation-dependent interaction with the SOCS3 SH2 domain and the reliance on an intact SOCS box for substrate ubiquitylation. Within the context of the JAK-STAT pathway, SOCS3 has been shown to bind Tyr-phosphorylated JAK1 and target it for degradation by the proteasome in a manner similar to how SOCS1 is thought to regulate JAK2 [29,37]. This section summarises other potential SOCS3 substrates and the biological impact of these events.

#### 3.2.1. Indoleamine 2,3-Dioxygenase (IDO)

Dendritic cells (DCs) initiate adaptive immune responses in part by presenting antigens to T cells via class II major histocompatibility complexes (MHC-II). However, specific DC subsets exist to facilitate a specific response. CD8^+^ DCs have a dual role in lymphoid tissue where, under stable conditions, they provide tolerance to self-antigen while becoming potent activators of CD8^+^ T cells upon infection [38]. IL-6-induced SOCS3 is sufficient to drive the switch from tolerogenic to immunogenic presentation of antigen peptides [35] and the enzyme indoleamine 2,3-dioxygenase (IDO), which is involved in tryptophan metabolism, is critical for this response. SOCS3 associates with IDO via an immunoreceptor tyrosine-based inhibitory motif (ITIM) site in a PTyr-dependent manner and catalyses its ubiquitylation and proteasomal degradation, thus clearing IDO and allowing an immunogenic response to progress. However, knockdown of SOCS3 results in up-regulation of IDO and a suppressed immunogenic CD8^+^ DC response that can be reversed with IDO inhibitor 1-MT [35]. Thus SOCS3 is critical for mobilising an effective immune response to infection.

#### 3.2.2. Focal Adhesion Kinase 1 (FAK1)

FAK1 is an essential, ubiquitously expressed, non-receptor protein tyrosine kinase that regulates foetal development, cell adhesion, migration, and survival. As such, upregulation of FAK1 is commonly associated with tumourigenicity and metastasis [39,40]. It does so by promoting glucose consumption, lipogenesis, glutamine dependency and regulating interactions with the extracellular matrix (ECM) (reviewed in [41]). Furthermore, hypermethylation of CpG islands within the SOCS3 promoter, which blocks SOCS3 induction, is detected in gliomas and hepatocellular tumours and can drive tumour cell migration through enhanced STAT3 and FAK1 activation [39,40]. Both SOCS3 and SOCS1, but not other SOCS family members, regulate FAK1 in a KIR- and SOCS box-dependent manner [30]. Integrin-activated FAK1 becomes autophosphorylated on PTyr397, enabling interaction with SOCS3 via its SH2 domain, and is subsequently inactivated via a KIR-dependent mechanism followed by Lys48-linked polyubiquitylation and proteasomal degradation. Interestingly, glucose uptake in endothelial cells is FAK1/insulin receptor substrate (IRS) 1-dependent, where IRS1 is a further SOCS3 E3 substrate (see Section 3.2.3). Thus SOCS3 might exert broad effects by targeting common effectors from a range of signalling pathways.

#### 3.2.3. Insulin Receptor Substrate (IRS) 1 and 2

Chronic inflammation and metabolic syndrome are linked to insulin resistance and type 2 diabetes mellitus. In each case, increased basal levels of cytokines leads to sustained, elevated levels of SOCS3, implicating SOCS3 in the development of these disorders [31]. In support of this hypothesis, Rui *et al.* [31] demonstrated that induction of both SOCS1 and SOCS3 in HEK293 and MCF7 breast cancer cells results in reduced levels of IRS1/2. This effect of SOCS1 was blocked following introduction of discrete Leu175Pro, Cys179Phe substitutions within the SOCS1 SOCS box, which prevented binding to the elongin B/C complex. However, these mutations had no impact on the ability of SOCS1 to bind IRS1/2, suggesting that the SOCS box is specifically required for down-regulation of IRS1/2 once SOCS proteins are bound [31]. Additionally, male C57BL/6 mice infected with SOCS1-expressing adenovirus, which regulates IRS1/2 similarly to SOCS3, resulted in a hyperglycaemic and insulin-resistant state which returned to a wild-type following loss of SOCS1. Interestingly, chronic hepatitis C virus (HCV) infection is also linked to type 2 diabetes mellitus [32] and increased fasting insulin levels are detected in HCV-infected patients. Kawaguchi *et al.* [32] found that SOCS3 is up-regulated in HCV-core transgenic mice liver or HCV-core-transfected human hepatoma cells. IRS1/2 were subsequently found to be polyubiquitylated and degraded by SOCS3, an effect that could be reversed by proteasome inhibitor MG132. Loss of IRS1/2 was also accompanied by inhibition of PI3K/Akt signalling which blocked glucose uptake. In addition, while HCV-core transfected WT mouse embryonic fibroblasts (MEFs) had reduced IRS1/2 levels, SOCS3^−/−^ MEFs were unaffected. Thus taken together, dysregulated SOCS3 expression appears to be important for the development of insulin resistance and type 2 diabetes mellitus.

#### 3.2.4. Sialic acid-binding Ig-like lectin (SIGLEC) 3 and 7

SOCS3 has so far been shown to exert mainly protective, anti-inflammatory effects under non-pathogenic conditions while also being involved in pro-inflammatory responses by regulating levels of T-cell and DC subsets [35,42,43]. SOCS3 might also have additional pro-inflammatory roles in sensitising myeloid cells to cytokines during inflammatory responses via degradation of the inhibitory receptors CD33/ SIGLEC 3 and SIGLEC 7 [33,34]. SIGLEC3/7 are inhibitory receptors expressed on myeloid cells and which, upon ligation with sialic acid-linked glycan agonists, can inhibit proliferation of myeloid cells. Orr *et al.* [33,34] have demonstrated that upon its induction, SOCS3 can bind SIGLEC3/7 at a phosphorylated Tyr340 ITIM and phosphorylated Tyr358 immunoreceptor tyrosine-based switch-like motif (ITSM), which is followed by degradation of both the receptor and SOCS3. Mutation of ITIM and ITSM sites or treatment with MG132 could protect both SIGLEC3/7 and SOCS3 from degradation. Similar to gp130, SHP1 and SHP2 share SOCS3 binding sites on SIGLEC3/7 [44], thus SHP-dependent signalling from SIGLEC3/7 could potentially be regulated by SOCS3.

#### 3.2.5. Granulocyte Colony-Stimulating Factor Receptor (G-CSFR)

Granulocyte colony-stimulating factor (G-CSF) is a cytokine that is critically involved in stimulating neutrophil production and, as such, is a key determinant of the innate immune system’s ability to mount an effective response against bacterial infections [45]. G-CSF mediates its effects though activation of a dimeric G-CSFR which activates the JAK-STAT pathway. Several studies have shown that G-CSF is able to trigger the induction of SOCS3 expression in model cell systems and polymorphonuclear neutrophils (PMNs), and that SOCS3 then interacts with the JAK-phosphorylated G-CSFR predominantly at Tyr729 [46]. Once bound, SOCS3 is thought to inhibit G-CSFR signalling by at least two distinct mechanisms: the first is through interaction of its KIR with receptor-bound JAKs via a process similar to that described for SOCS3 inhibition of gp130 [16,17]. The second is through regulation of G-CSFR trafficking into lysosomal compartments for degradation after ligand-induced internalisation [36,47]. Interestingly, ubiquitylation of Lys632 in the G-CSFR by SOCS3 appears to be critical for receptor sorting rather than proteasomal degradation, as a Lys632Arg mutant receptor fails to co-localise with Rab7-positive lysosomal compartments following G-CSF-mediated internalisation. Crucially, mutation of either Lys632Arg (site of ubiquitylation) or Tyr729Phe (SOCS3 binding site) results in sustained STAT5a activation and enhanced G-CSF-stimulated proliferation responses compared with the WT receptor, suggesting that SOCS3-mediated ubiquitylation of Lys632 and subsequent lysosomal degradation of the receptor is a critical mechanism by which G-CSFR signaling is terminated [36]. Consistent with this hypothesis, previous studies using mice expressing a C-terminally truncated SOCS3 that lacks the SOCS box demonstrated that loss of this domain resulted in sustained G-CSF-stimulated STAT3 activation, a hypersensitivity of bone marrow-derived cells to G-CSF in proliferation assays and a more severe joint pathology in an acute model of arthritis [27]. However the nature of the ubiquitin chain(s) attached to Lys632 remains unknown, although it is unlikely to include Lys48 linkages given the lack of involvement of the proteasome in this process. Interestingly, Wölfler *et al.* [47] have noted that Lys residues in a similar juxta-membrane location are found in several cytokine receptors, including SOCS3 targets gp130 and ObRb, although gp130 appears to constitutively internalize and then degrade independent of IL-6 stimulation following interaction of AP-2 with a cytoplasmic di-Leu motif [48,49].

Taken together these data support an important role for the ECS^SOCS3^ E3 ubiquitin ligase in regulating a wide range of biological functions. Moreover, they also demonstrate that dysregulation of this aspect of SOCS3 function may be integral to the development of several disorders, including diabetes, cancers and multiple chronic inflammatory and immune diseases.

## 4. Regulation of SOCS3 Turnover

### 4.1. Introduction

While induction of the SOCS3 gene by multiple diverse stimuli has been well characterised, much less is known about post-translational regulation of SOCS3 function. What is clear is that SOCS3 can be rapidly polyubiquitylated, a process which targets the protein for degradation by the proteasome. Consequently, SOCS3 has a relatively short biological half-life, ranging from 40–120 min depending on the cell type under investigation [50,51]. One study in Ba/F3 pro-B cells has determined that Lys6 may be critical for SOCS3 ubiquitylation and subsequent degradation by the proteasome [51], although it has since been demonstrated that the PEST sequence within the SH2 domain (Figure 2) is also a key regulator of SOCS3 stability that functions independently from the proteasome [52]. Therefore the relative importance of these distinct mechanisms may vary between cell types.

### 4.2. SOCS3 Tyr Phosphorylation and Degradation

Ubiquitylation is not the only post-translational modification that may regulate SOCS3 stability. For example, phosphorylation of Tyr204 and Tyr221 within the BC box sub-domain of the SOCS box region has been shown to enhance SOCS3 degradation via the proteasome [53]. Induction of endogenous SOCS3 expression in RAW264 monocytic cells via LPS exposure revealed a significant increase in SOCS3 protein levels that was blocked upon the addition of the Tyr phosphatase inhibitor sodium orthovanadate, which sustained SOCS3 phosphorylation on Tyr204 and Tyr221. Haan and colleagues went on to show that following Tyr204/221 phosphorylation, the interaction between SOCS3 and elongin C was abolished, therefore suggesting a role for the SOCS box-elongin C interaction in stabilising SOCS3 [53]. Further characterisation demonstrated that upon IL-6 stimulation, SOCS3 phosphorylation on Tyr204 and Tyr221 is JAK-independent and is instead driven by either Src or receptor tyrosine kinases [54]. However it has been proposed that a Val617Phe-mutated constitutively active JAK2 mutant associated with several myoproliferative disorders, including polycythemia vera, can interact with SOCS3 in a distinctive fashion. This has several consequences: firstly, it is associated with an inability of SOCS3 to inhibit Val617Phe JAK2 activity. In fact, SOCS3 actually appeared to *enhance* the proliferative effects of erythropoietin (EPO) in stably transfected Ba/F3 cells stably co-expressing EPO receptors and Val617Phe JAK2 while inhibiting EPO-stimulated proliferation in cells co-expressing EPOR and WT JAK2 [55]. Secondly, Val617Phe JAK2 expression promoted a robust increase in Tyr phosphorylation of SOCS3 which, in contrast to previous findings [53], actually stabilised SOCS3 protein levels by an unknown mechanism [55]. However the situation is likely more complex, as in a separate study others have found that under different conditions SOCS3 is capable of inhibiting the activity of co-expressed Val617Phe JAK2, while siRNA-mediated knockdown of both SOCS3 and SOCS1 could enhance Val617Phe JAK2 protein levels [56].

### 4.3. SOCS2 as a Regulator of SOCS3 Stability and Turnover

Two studies have independently proposed that turnover of SOCS3 can be controlled by SOCS2 acting as an ECS^SOCS2^ E3 ligase complex [57,58]. Analysis of SOCS expression in a variety of cell types by Tannahill *et al.* [57] revealed a reciprocal regulation of SOCS2 expression on SOCS3 levels, resulting in impaired SOCS3-mediated suppression of STAT3 and STAT5b activation and cell proliferation by IL-3 when SOCS2 levels are enhanced [57]. Further characterisation of this phenomenon in transfected cells demonstrated that SOCS2-mediated proteasomal down-regulation of SOCS3 required the SOCS2 SOCS box domain and was enhanced by co-expression of elongins B and C. The capacity of SOCS2 to suppress the inhibitory effect of SOCS3 (and other SOCS family members) on cytokine signalling required the SOCS2 SOCS box, and SOCS2 was also shown to stimulate the rapid turnover of co-expressed SOCS1. Together, these data suggest that SOCS2 binding to other SOCS family members can accelerate their turnover by a mechanism that requires elongin B/C recruitment via a functional SOCS box and therefore may trigger their ubiquitylation and degradation by the proteasome [58]. While these studies have been limited to *in vitro* analysis of recombinant SOCS proteins in model cell systems, the mechanisms described provide an attractive explanation for the reciprocal effects of SOCS2 and SOCS3 on macrophage polarisation observed by Spence *et al.* [59]. However work carried out in macrophages from SOCS2^-/-^ mice has shown that the kinetics of SOCS3 accumulation and SOCS3 functionality are not altered by the absence of SOCS2. Importantly, neither the induction nor decay of SOCS3 expression was altered by SOCS2 deletion, although the authors failed to examine whether SOCS3 turnover in these cells was via a proteasome-dependent mechanism or whether alternate pathways involving the SOCS3 PEST domain were involved [60].

## 5. Future Directions

The significance of unresolved inflammatory and immune responses driven by the JAK-STAT pathways in a variety of pathologies, including myeloproliferative disorders, rheumatoid arthritis, Crohn’s Disease and also atherosclerosis, is now well established. Indeed, several therapeutics targeting the JAK-STAT pathway, including IL-6R-targeted antibodies and small molecule JAK inhibitors, are now available for some of these conditions, with several more in late stage development [61,62]. Exploiting the various inhibitory mechanisms invoked to limit IL-6 signalling therapeutically with the aim of generating small molecules capable of either arresting or reversing disease progression is now an important goal. Progress in understanding the molecular mechanisms underlying the regulation and function of SOCS3 will undoubtedly help inform these approaches.

What makes SOCS3 of particular interest therapeutically is its capacity to suppress the activity of multiple intracellular targets. In this regard it may be worth exploring the potential application of manipulating SOCS3 expression and/or function in the many diseases for which localised inflammation is only one aspect of the developing pathology, including acute vascular injury scenarios such as coronary artery bypass grafting and percutaneous coronary intervention (PCI). PCI is a re-vascularisation procedure which typically involves implantation of a stent into narrowed coronary artery to reduce the risk of myocardial infarction [63]. A stent is essentially a metallic mesh cage that physically holds open the previously narrowed blood vessel lumen, thereby restoring blood flow. However, mechanical injury occurs during this procedure, causing the release of pro-inflammatory and mitogenic factors such as tumour necrosis factor α (TNFα) and insulin-like growth factor-I (IGF-I). Stimulation of human and porcine coronary artery smooth muscle cells (SMCs) with TNFα or IGF-I alone up-regulates SOCS3 while co-stimulation with both TNFα and IGF-I leads to a decrease in SOCS3 transcription in these cells [64,65]. Interestingly, pre-treatment of vascular SMCs with transcriptional inhibitor actinomycin D confirmed that TNFα and IGF1 stimulation inhibited SOCS3 mRNA synthesis, and that this was associated with formation of PTyr705STAT3/RelA complexes in the nucleus. Additionally, hypermethylation of the CpG island in the SOCS3 promoter by DNA methyltransferase-I (DNMT1) was observed in TNFα and IGF-I-stimulated human coronary artery SMCs, revealing a conserved epigenetic mechanism by which SOCS3 expression could be repressed [65]. From these observations, it has been proposed that silencing of SOCS3 expression contributes to disease progression via a loss of inhibitory control of JAK-STAT signalling in coronary artery SMCs which may drive neo-intimal lesion formation by increasing STAT activation and target gene expression. Key STAT target genes include cell cycle regulator cyclic D1 and matrix metalloproteases MMP-2 and MMP-9 [66,67,68], which degrade extracellular matrix and other proteins to trigger vascular re-modelling and blood vessel re-narrowing. By virtue of its activity as part of an ECS^SOCS3^ E3 ubiquitin ligase complex, a key target of SOCS3 could be FAK1, which has been shown to control integrin-stimulated vascular SMC migration [69].

Thus, in the context of either in-stent re-stenosis, localised accumulation of SOCS3 would be anticipated to suppress endothelial inflammation (via inhibition of IL-6 signalling), vascular smooth muscle cell proliferation (via inhibition of STAT3 activation), migration (via inhibition of FAK1) and also re-modelling (via reduced induction of MMP-2 and MMP-9) [66,67]. Local elevation of SOCS3 expression *in vivo* has already been shown to have beneficial effects on limiting inflammatory cell infiltration and the ensuing tissue dysfunction in mouse models of specific inflammatory diseases, including systemic bacterial infection, hepatitis, and rheumatoid arthritis [70,71]. The advent of drug-eluting and bio-absorbable polymer eluting stents for PCI provides an obvious route through which strategies to elevate SOCS3 expression could be deployed locally at the site of injury in the coronary artery, thereby minimizing the possibility of SOCS3 accumulation in non-diseased tissues. For example, small molecule DNMT1 inhibitors could be used to de-repress epigenetic suppression of SOCS3 gene transcription, thereby enhancing SOCS3 expression to limit endothelial inflammation (via inhibition of IL-6 signalling) as well as vascular SMC proliferation (via inhibition of STAT3 activation), migration (via inhibition of FAK1) and re-modelling (via reduced induction of STAT-regulated genes MMP-2 and MMP-9). However, as SOCS3 is turned over quite rapidly (see Section 4.1) by both proteasome-dependent mechanisms as well as a proteasome-independent route involving the PEST sequence (see Section 4.1), optimal therapeutic efficacy targeting SOCS3 may require combined inhibition of both transcriptional repression and turnover. Alternatively, stents could be utilized to deliver alternate therapies to the injured vessel aimed at increasing SOCS3 expression to limit re-stenosis. Approaches to achieve this have included administration of recombinant SOCS3 adenovirus [70] or purified SOCS3 protein modified with a membrane-translocating motif from a hydrophobic signal sequence derived from fibroblast growth factor 4 to confer cell permeability [71]. As proof of concept, preliminary studies already suggest that SOCS3 gene therapy is effective in reducing neointimal hyperplasia in a rat vein grafting model, and can inhibit aortic SMC inflammation, migration and proliferation responses *in vitro* [72]. The identification of E3 ligases controlling SOCS3 ubiquitylation and degradation may give rise to the development of specific inhibitors or peptide disruptors that could ultimately stabilise the expression of SOCS3 in the vasculature. Similarly, identifying the key Lys residues regulating SOCS3 turnover may facilitate the engineering of a mutated SOCS3 that cannot be ubiquitylated and is, therefore, more stable and able to provide a more sustained inhibition of re-stenosis.

Testing these kinds of approaches in additional disease models, coupled with the development of strategies to generate small molecules capable of triggering the accumulation of SOCS (e.g., inhibitors of E3 ubiquitin ligases that target SOCS3 for destruction), will allow an assessment of whether the potential for such approaches can be realised in a range of therapeutic indications other than those with a strong pro-inflammatory component.
